# From network biology to immunity: potential longitudinal biomarkers for targeting the network topology of the HIV reservoir

**DOI:** 10.1186/s12967-025-06919-z

**Published:** 2025-08-13

**Authors:** Heng-Chang Chen

**Affiliations:** Quantitative Virology Research Group, Population Diagnostics Center, Łukasiewicz Research Network – PORT Polish Center for Technology Development, Stablowicka 147, 54-066 Wrocław, Poland

**Keywords:** Network topology, Network analysis, Graph theory, Biological networks, Deep learning, HIV reservoir, Immunologic signatures, Elite controllers, Longitudinal biomarkers

## Abstract

In the “omics” era, studies often utilize large-scale datasets, eliciting the overall functional machinery of a network’s organization. In this context, determining how to read the enormous number of interactions in a network is imperative to comprehend its functional organization. Topology is the principal attribute of any network; as such, topological properties help to elucidate the roles of entities and represent a network’s behavior. In this review, I showcase the foundational concepts involved in graph theory, which form the basis of network biology, and exemplify the application of this conceptual framework to bridge the connection between the task-evoked functional genome network of the HIV reservoir. Furthermore, I point out potential longitudinal biomarkers identified using network-based analysis and systematically compare them with other potential biomarkers identified based on experimental research with longitudinal clinical samples.

## Introduction

Latent HIV reservoir cells harboring persistent proviruses are one of the main obstacles to curing HIV; this is due to the awakeness of latent proviruses when treatment is interrupted. The uncertainty of the precise location of reservoir cells harboring latent proviruses renders antiretroviral therapy (ART) inefficient. Scientific advances have been made in relation to omics and tailored approaches, including single-cell RNA sequencing, full-length individual proviral sequencing, and profiling a pattern across an abundant number of single cells, facilitating the localization of reservoir cells that are separated by intact and defective proviruses. In addition, the identification of potential phenotypic signatures and cell markers targeting reservoir cells is also critical for HIV therapeutics. More recently, our laboratory proposed the use of genomic approaches associated with mathematics to represent the HIV reservoir as a functional genome property of a network during the course of HIV infections associated with ART [1–3], aiming to unveil new features and variables for fine-tuning the microenvironment of the HIV reservoir [3].

Indeed, an increasing number of studies have revealed that the establishment of HIV reservoirs is a dynamic progression, whereby the presence of the indicators is not consistent with disease progression and the expression varies between the different stages of HIV infection. This observation implies that the longitudinal tracking of disease progression in patients is thus imperative to better characterize the latent HIV reservoir; additionally, a series of longitudinal biomarkers appearing in different stages during the course of infection will be requisite to recapitulate the reservoir cells where persistent infections occur entirely. In this Review, I categorize potential longitudinal biomarkers into five groups, including (1) HIV [4–17], (2) immunity (including immune cells and proinflammatory factors) [1, 10, 18–34], (3) cellular molecules and soluble factors [9, 18–21, 26, 28, 31, 34–42], (4) host genome/factors [30, 43, 44], and (5) epigenomes [45, 46], based on the research articles from the last five years (2019–2024) (Table 1), comparing them to the features identified based on network-based approaches [1, 3].

**Table 1:** Systematic summary of the research articles, in which potential longitudinal biomarkers targeting the HIV reservoir were proposed/identified

Title	References	Samples	Longitudinal measurement	Category of longitudinal biomarkers	Longitudinal biomarkers	Finding
Impact of interrupting antiretroviral therapy started during primary HIV-1 infection on plasma neurofilament light chain protein, a marker of neuronal injury: The SPARTAC trial	Alagaratnam et al. [31]†	Plasma from participants enrolled in SPARTAC [121]	Weeks 0 (before starting ART), 48 (after 48 weeks of ART), and 60 (12 weeks after stopping ART)	Cellular molecules & soluble factors; Immunity	NfL; IL-6	A decrease of plasma NfL in neuro-axonal injury in patients following ART initiation during primary HIV infection was observed
Early antiretroviral therapy and its impact on natural killer cell dynamics in HIV-1 infected men who have sex with men: a cross-sectional pilot study evaluating the impact of early ART initiation on NK cell perturbation in HIV infection	Akiso et al. [32]	Blood samples	Pre-infection and sequential bi-weekly post-infection upto 30 days	Immunity	NK cells	Early initiation of ART may be important in boosting innate immunity and restoring some perturbations of NK cells
Accelerated CD8 + T cell maturation in infants with perinatal HIV infection	de Armas et al. [42]‡	Peripheral blood sample	Multiple timepoints throughout the study before and after starting ART	Cellular molecules & soluble factors	CD38^+^HLADR^+^CD8 T cells	CD38^+^HLADR^+^CD8 T cells are a strong correlate of virus load in perinatal HIV
Longitudinal patterns of inflammatory mediators after acute HIV infection correlate to intact and total reservoir	De Clercq et al. [33]	Plasma samples	T0: before ART initiation (T0); DVL: at decreasing viral load on ART; UD: after suppression of plasma viral load; UD + 1: one year later after suppression of plasma viral load	Immunity	Various factors, see Figs. 3 and 5	Various immune cells and the abundance of proinflammatory factors correlate with the course of acute HIV infection
HIV-1 RNA in extracellular vesicles is associated with neurocognitive outcomes	DeMarino et al. [17]†	Extracellular vesicles from CSF/serum	43 HIV infected individuals with two or more visits	HIV	HIV RNA	Extra-cellular vesicles-associated viral RNA was more abundant in the cerebrospinal fluid (CSF) and correlated with neurocognitive dysfunction in the cross-sectional and longitudinal studies
Observational study of effects of HIV acquisition and antiretroviral treatment on biomarkers of systemic immune activation	Kosmider et al. [34]	Four longitudinally collected plasma	Before infection and after 2 years of effective ART	Cellular molecules & soluble factors; Immunity	CRP, MCP-1/CCL2, leptin, LBP, IP-10; TNF-alpha	The levels of CRP, MCP-1/CCL2, TNF-α and IP-10 significantly increased while leptin and LBP significantly decreased following HIV infection across 47 patients
HIV RNA/DNA levels at diagnosis can predict immune reconstitution: a longitudinal analysis	Basoulis et al. [13]	Peripheral blood mononuclear cell (PBMC) samples	The first 3 months after cART initiation	HIV	HIV DNA and RNA	Higher DNA and RNA levels correlate with greater CD4 increase past the first trimester
The largest HIV-1-infected T cell clones in children on long-term combination antiretroviral therapy contain solo LTRs	Botha et al., 2023 [14]‡	Blood samples	Specimens were collected from participants with HIV early and post antiretroviral cohort study	HIV	Defective proviruses with solo LTR	Solo LTRs comprise a large fraction of the proviruses in infected cell clones that persist in children on long-term cART
Machine Learning Bolsters Evidence That D1, Nef, and Tat Influence HIV Reservoir Dynamics	Cannon et al. [15]	Longitudinal near full-length sequences	7 people living with HIV between 1 and 20 years following the initiation of antiretroviral treatment	HIV	The splice donor site D1, HIV *nef* and *tat*	D1: reservoir contraction. HIV *nef*: a protective advantage for latently infected T cells. HIV *tat*: is associated with clonal expansion
The Dynamic Linkage between Provirus Integration Sites and the Host Functional Genome Property Alongside HIV-1 Infections Associated with Antiretroviral Therapy	Chen [1]	Published data retrieved from Jiang et al. [101] and Einkauf et al. [103]	Pretreatment; patients subjected to a short (less than 1 year) and long (more than 1 year) of ART	Immunity	see Figs. 3 and 5	HIV integration frequency might be used as a surrogate for gene sets, which may define specific immune cell types and proinflammatory soluble factors during HIV infection
Plasma proteomic profiling identifies CD33 as a marker of HIV control in natural infection and after therapeutic vaccination	Duran-Castells et al. [38]	Plasma samples	Participants completed the 32 weeks of monitored antiretroviral pause	Cellular molecules & soluble factors	CD33/ Siglec-3	CD33/Siglec-3 discriminates between MAP-C and MAP-NC
Sirtuin-2, NAD-dependent deacetylase, is a new potential therapeutic target for HIV-1 Infection and HIV-related neurological dysfunction	Duran-Castells et al. [39]†	Plasma samples	Immediate or delayed initiation (after 1 year) of cART	Cellular molecules & soluble factors	Sirtuin-2	Sirtuin-2 is strongly associated with elevated viral loads and HIV provirus levels
Viral and host mediators of non-suppressible HIV-1 viremia	Mohammadi et al. [30]	Plasma samples	8–12 years post ART	Genome; immunity	H3K36me3; HIV-specific CD8 T cell responses	Integration sites of producer proviruses were enriched in proximity to the activating H3K36me3. Participants with NSV showed significantly lower HIV-specific CD8 T cell responses
Longitudinal characterization of circulating extracellular vesicles and small RNA during simian immunodeficiency virus infection and antiretroviral therapy	Huang et al. [40]	Extracellular vesicle enriched blood plasma samples	Samples harvested during preinfection, acute infection, latent infection/ART treatment, and rebound after ART interruption	Cellular molecules & soluble factors	Cellular small RNAs	Extracellular vesicle small RNA expression correlates with viral load and the course of HIV infection
Predictors of intact HIV DNA levels among children in Kenya	Neary et al. 2023 [16]‡	PBMC samples	12, 24, 42, 60, and 96 months post-ART initiation	HIV	Intact HIV DNA	Pre-ART immunosuppression, first-line ART regimen, and the viral load of cytomegalovirus may influence establishment and sustainment of intact HIV DNA in the reservoir
Progressive transformation of the HIV-1 reservoir cell profile over two decades of antiviral therapy	Lian et al. [44]	PBMC samples	Time span over years	Genome	Heterochromatin	Persistent infections of intact proviruses in heterochromatin
HLA-B*57 and B*58 associate with predictors of reservoir size in an acutely treated HIV cohort	Shangguan et al. [41]	Plasma samples	Before ART initiation and 24 weeks after starting therapy	Cellular molecules & soluble factors	HLA-B*57 & B*58	HLA-B*57 and B*58 are associated with time to viral load suppression, which was one of the predictors of the size of the HIV reservoir
Cellular Activation, Differentiation, and Proliferation Influence the Dynamics of Genetically Intact Proviruses Over Time	Horsburgh et al. [37]	PBMC samples	Two time points (leukapheresis 1 and 2) during ART	Cellular molecules & soluble factors	HLA-DR	Cellular mechanisms such as activation, differentiation, and proliferation influence the dynamics of the HIV reservoir
HIV-1 Evolutionary Dynamics under Nonsuppressive Antiretroviral Therapy	Kemp et al. [11]	Plasma samples	Multiple time points across the first and second regimens	HIV	Mutations in reverse transcriptase	Drug resistance mutations in reverse transcriptase were used as markers of viral haplotypes in the reservoir
Natural killer cell receptors and ligands are associated with markers of HIV-1 persistence in chronically infected ART suppressed patients	Ivison et al. [27]	PBMC samples	3 on-ART timepoints with a median of 7 years	Immunity	NK cells	NK cell ligands CD58, HLA-B, CRACC, and the killer cell immunoglobulin-like receptors KIR2DL1, KIR2DL3, and KIR2DS4 are predictive of markers of HIV-1 persistence
The role of CD101-expressing CD4 T cells in HIV/SIV pathogenesis and persistence	Strongin et al. [28]	Peripheral blood, lymph node and rectal biopsy samples	Acute (days 14–18 post-infection), early chronic (days 42–56 post-infection), early ART (6 weeks post-ART initiation), mid ART (24 weeks post-ART initiation), late ART (33–60 weeks post-ART initiation)	Immunity	CD101 Treg	CD101^+^CD4 T cells present at all stages of HIV/SIV infection
Two distinct mechanisms leading to loss of virological control in the rare group of antiretroviral therapy-naive, transiently aviremic children living with HIV	Vieira et al. [29]‡,#	PBMC samples	Several time points over years	Immunity	HIV-specific CD8 T cell response	HIV-specific CD8 T cell response can achieve viremic control without viral escape
Complex decay dynamics of HIV virions, intact and defective proviruses, and 2LTR circles following initiation of antiretroviral therapy	White et al. [12]	PBMC samples	Samples were collected before the initiation of ART, every 2 weeks for the first 3 moths, and once a month thereafter for up to a year	HIV	Intact and defective proviruses, and 2LTR circles	A rapid decay of intact proviruses in circulating CD4 T cells compared with defective proviruses; 2LTR circles decay with fast and slow phases paralleling intact proviruses
Candidate host epigenetic marks predictive for HIV reservoir size, responsiveness to latency reversal, and viral rebound	Corley et al. [46]	Purified CD4 T cells	Time span on ART and after ART	Epigenome	DNA methylation	A distinct set of 15 candidate DNA methylation sites in purified CD4 T cells correlated with time to viral rebound
Restriction Factors expression decreases in HIV-1 patients after cART	D'Urbano et al. [43]	Blood samples	T0: at the time of HIV diagnosis before starting antiretroviral treatment; after 4 (T1) and 8 (T2) months from the beginning of cART from patients	Host factors	Restriction factors	Expression of restriction factors is correlated with viral replication
Selective Decay of Intact HIV-1 Proviral DNA on Antiretroviral Therapy	Gandhi et al. [6]	Blood samples	From a median of 7.1 years to 12 years after ART initiation	HIV	HIV genome integrity	Intact provirus levels on ART correlated with total HIV DNA and residual plasma viremia
Heightened resistance to host type 1 interferons characterizes HIV-1 at transmission and after antiretroviral therapy interruption	Godim et al. [23]	Plasma samples	Time span over 12.4 years	Immunity	IFN-1	Resistance of HIV isolates to IFN-I was high during acute infection, decreased in the first year after infection, and remained elevated in individuals with accelerated disease
Long-term effects of early antiretroviral initiation on HIV reservoir markers: a longitudinal analysis of the MERLIN clinical study	Massanella et al. [7]	PBMC samples	Samples collected before ART initiation and longitudinally for up to 4 years on ART	HIV	Total and integrated HIV DNA	A biphasic decay of all HIV persistence markers, with a rapid initial decline followed by a slower decay in all participants
Naive infection predicts reservoir diversity and is a formidable hurdle to HIV eradication	Pinzone et al. [36]#	PBMC samples	Samples collected over time since diagnosis	Cellular molecules & soluble factors	CD45RA^+^CCR7^+^CD27^+^CD95^–^ naive cells	Naive cells play a critical role in HIV persistence, predicting reservoir size and diversity
Cell-associated HIV-1 unspliced-to-multiply-spliced RNA ratio at 12 Weeks of ART predicts immune reconstitution on therapy	Scherpenisse et al. [8]	PBMC samples	At 0, 12, 24, 48, and 96 weeks of virologically suppressive ART	HIV	Cell-associated HIV unspliced-to-multiply-spliced (US/MS) RNA	A higher US/MS RNA ratio may reflect the higher frequency of productively infected cells that could exert pressure on the immune system
Cerebrospinal fluid CD4 + T cell infection in humans and macaques during acute HIV-1 and SHIV infection	Sharma et al. [9]†	PBMC, lymph node mononuclear cells, and CSF cell pellet samples	Macaques: post-infection sampling occurred at weeks 4, 8, and 12, with additional PBMC collection 2–3 weeks; humans: fiebig stages III and IV (an estimated 14 and 19 days post-infection, respectively)	HIV; Cellular molecules & soluble factors	SHIV RNA; CD38, IP-10	Surface markers of activation were increased on CSF T cells and monocytes and correlated with CSF soluble markers of inflammation
HIV-specific T cell responses reflect substantive in vivo interactions with antigen despite long-term therapy	Stevenson et al. [24]	PBMC samples	Samples collected at 24 weeks and 168 weeks after a median of 7 (range 4–15) years after ART initiation	Immunity	HIV Nef-specific T cell responses	The rates of change in persisting HIV Nef-specific T cell responses were associated with residual frequencies of infected cells
Anti-Tat immunity defines CD4 + T-cell dynamics in people living with HIV on long-term cART	Tripiciano et al. [25]	PBMC samples	Time span over 3 years	Immunity	CD8 T cells, B cells	CD4 T-cell restoration reflects the interplay among HIV Tat immunity, residual viremia and immunological determinants including CD8 T cells and B cells
Therapeutic prediction of HIV-1 DNA decay: a multicenter longitudinal cohort study	Yue et al. [10]	PBMC samples	Time span from 0 to 288 weeks	HIV; Immnity	HIV DNA; CD4 T cell count	Baseline total HIV DNA and CD4 T cell count are two independent predictors of total HIV DNA after treatment
Dynamics of HIV reservoir decay and naïve CD4 T-cell recovery between immune non-responders and complete responders on long-term antiretroviral treatment	Zhang et al. [26]	PBMC samples	Samples collected at years 1, 3 and 5 of ART	Cellular molecules & soluble factors; Immunity	PD-1^+^CD4 T-cells, naïve CD4 T-cells	Immunological non-responders maintained higher levels of HIV DNA and cell-associated-RNA with higher percentages of PD-1^+^CD4 T cells compared with complete responders during 5-year ART, concurrent with lower naïve CD4 T cells
Activated PD-1 + CD4 + T cells represent a short-lived part of the viral reservoir and predict poor immunologic recovery upon initiation of ART	Eller et al. [21]	PBMC samples	donors at baseline and at 6 and 12 months on ART	Cellular molecules & soluble factors; Immunity	PD-1 CD4 T cells	Activated PD-1 CD4 T cells are predictors of poor immunologic recovery on ART and may represent a short-lived component of HIV reservoirs
Levels of Human Immunodeficiency Virus DNA Are Determined Before ART Initiation and Linked to CD8 T-Cell Activation and Memory Expansion	Martin et al. [35]	Cell population	Time span across the period of pretreatment and ART	Immunity	see Figs. 3 and 4	Pre-ART HIV DNA is significantly associated with various types of immune cells
Persistent expansion and Th1-like skewing of HIV-specific circulating T follicular helper cells during antiretroviral therapy	Niessl et al. [22]	Blood CD4 T cells	Time span across the pretreatment and after ART	Immunity	Circulating Tfh cells	Distinct HIV-specific circulating Tfh profile was induced during chronic untreated HIV infection, persisted on ART and correlated with the translation-competent HIV reservoir
HIV-1 diversity and compartmentalization in urine, semen, and blood	Stadtler et al. [5]	Urine, semen, and blood samples	At weeks 0, 2, 12, and 20 post-enrollment	HIV	HIV *env* sequences	Different viral kinetics between the upper and lower genitourinary tract
Determinants of HIV-1 reservoir size and long-term dynamics during suppressive ART	Bachmann et al. [4]	PBMC samples	1.5 ± 0.5 years, 3.5 ± 0.5 years, and 5.5 ± 1 years ater initiating first-line ART	HIV	Viral blips, low-level viremia	Viral blips and low-level viremia are significantly associated with slower reservoir decay
Longitudinal variation in human immunodeficiency virus long terminal repeat methylation in individuals on suppressive antiretroviral therapy	Cortés-Rubio et al. [45]	Total CD4 T cells	Samples obtained on short-term ART (30 months) followed up for 2 years	Epigenome	HIV 5'LTR methylation	HIV 5'-LTR methylation in total CD4^+^ T cells is dynamic along time
Early antiretroviral therapy in neonates with HIV-1 infection restricts viral reservoir size and induces a distinct innate immune profile	Garcia-Broncano et al. [18]‡	PBMC samples	Neonates with HIV infection started ART shortly after birth and were followed longitudinally for about 2 years	Cellular molecules & soluble factors; Immunity	see Figs. 3 and 4	Natural killer cells and other components of the innate immune system are critical for host immune protection in the postnatal period of infants with HIV infection
Memory CD4 + T-cells expressing HLA-DR contribute to HIV persistence during prolonged antiretroviral therapy	Lee et al. [19]	PBMC samples	Time span over over 3 to ≥ 15 years of effective therapy	Cellular molecules & soluble factors; Immunity	see Figs. 3 and 4	Distinguishable cellular activation/exhaustion markers between HLA-DR^−^ and HLA-DR^+^ memory CD4 T cell subsets
Rapid development of HIV elite control in a patient with acute infection	Morley et al. [20]#	Purified peripheral blood CD4 T cells	6 samples between December 2015 and January 2017 (before and after ART initiation)	Cellular molecules & soluble factors; Immunity	see Figs. 3 and 4	The patient subsequently developed detectable anti-HIV antibodies and an increase in HIV-specific CD8 T cell responses to overlapping subtype C HIV *gag* peptide

SPARTAC: Short Pulse Anti-Retroviral Therapy at Seroconversion [121]; NSV: Non-suppressible HIV viremia; MAP-C: Monitored antiretroviral pause (MAP-C, ‘controllers’); MAP-NC: Monitored antiretroviral pause (MAP-NC, ‘non-controllers’)

^†^Studies related to the HIV reservoir in the central nervous system

^‡^Studies related to infants

^#^Studies related to elite controllers

This review is structured into three parts. In the first part, I briefly introduce foundational but essential concepts in graph theory. I start by explaining what a graph is and how it can be seen as a network. Furthermore, I emphasize an argument that is focused on why topology matters while investigating a network’s properties, highlighting critical methodologies that are used to characterize its topology. Thirdly, I strengthen the potential application of a theoretical graph network to a real-world network, focusing on how to properly learn biological information from a network. In the “Outstanding questions” Section, I briefly discuss the dynamics of a network and its evolution, emphasizing *Markovian stochastic processes*. To facilitate reading, I offer a glossary related to graph theory (which includes all terms written in *italics*) at the end of this article. The second part describes a collection of human immunodeficiency virus (HIV)-related studies in which network-based analyses have been conducted. One of the aims of this part is to underscore the intrinsic difference in reservoirs between non-controllers and elite controllers (who can spontaneously suppress HIV replication without treatments), leading us to introduce the difference in the evolution of graph networks between these two types of HIV-infected individuals. In the third part, the emphasis is placed on the discussion of the potential longitudinal biomarkers that are responsible for the different stages of HIV infections, highlighting those that can also be identified using network-based approaches [1, 3]. At the end of this article, I illustrate my viewpoint about the potential application of network topology, representing the HIV reservoir for therapeutic regimens tailored to individual patients.

## The principle of a network

### What is a network?

A network is a collection of functionally interdependent and structurally interconnected components comprising an integrated whole. It arranges the distribution of individual entities based on their correlations or similarity, where are measured according to the features showcased by a property. Thus, graph theoretical tools provide a means to visualize relationships across individual entities in a network.

**Fig. 1:**
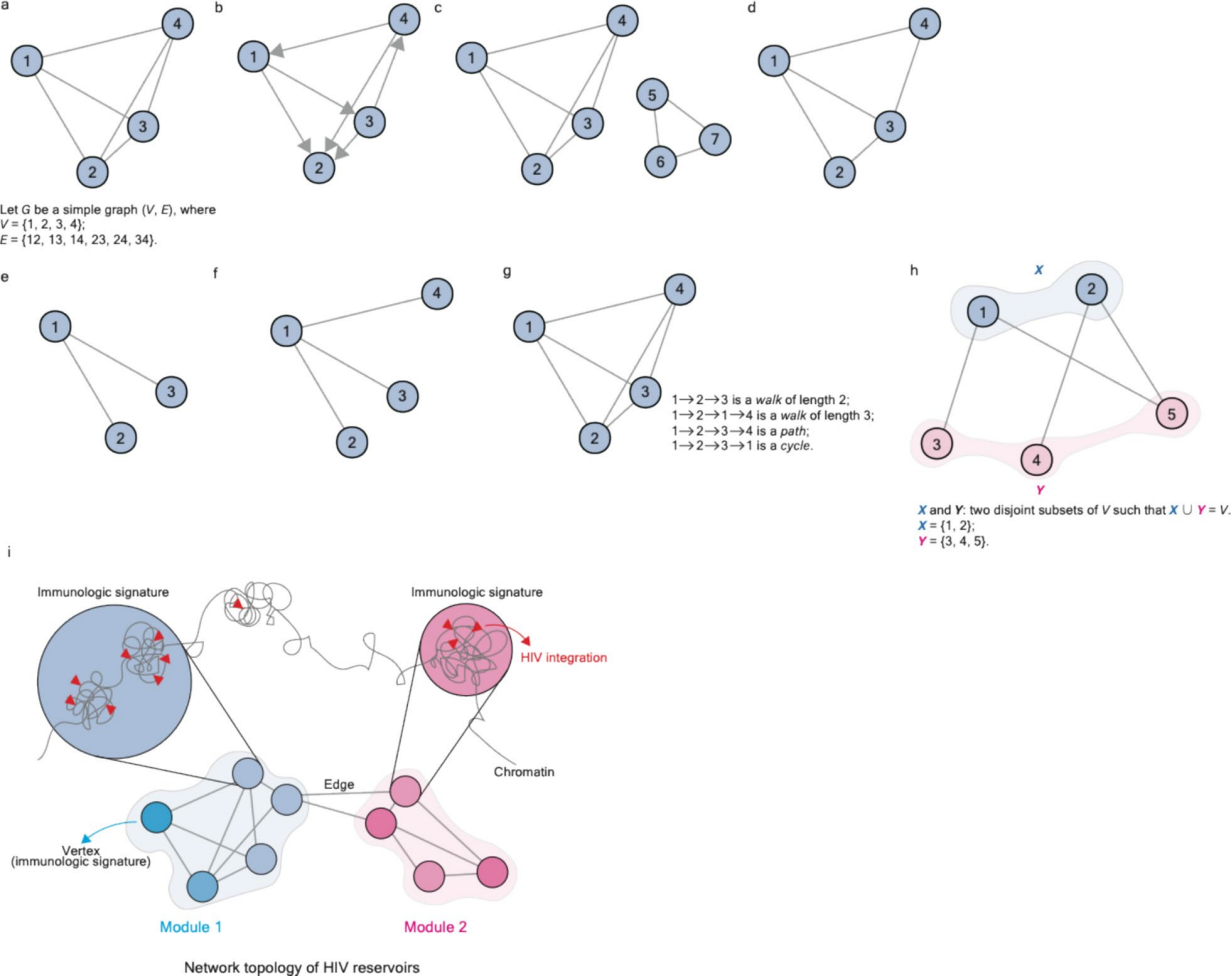
Schematic illustration of different forms of a graph. **a** A *simple graph*. It is composed of *vertices* (*V*) and *edges* (*E*). A *V* set includes the *vertices* 1, 2, 3, and 4; an *E* set includes the *edges* 12, 13, 14, 23, 24, and 34. **b** A *directed graph*. Directions, represented by arrowheads, were assigned to the *edges*. **c** A *disconnected graph*. No *path* is connected between a *V* set, 1, 2, 3, and 4, and another set, 5, 6, and 7. **d** A *planar graph*. **e** A *forest*, **f** A *tree*. **g** A *simple graph*, illustrating *a walk*, *a path*, and a cycle. **h** A *bipartite graph* that consists of two disjoint *V* sets, X labeled in blue and Y labeled in magenta. Of note, definitions, and descriptions corresponding to each form of the graph were detailed in the main text and the *Glossary*. **i** Schematic illustration of the hypothesis of the topological network representing HIV reservoirs. In our studies, we hypothesize that HIV-targeted genes (represented by red triangles shown in the cartoon) harboring similar biological functions related to immunity can form different gene sets, namely “immunologic signatures”, to satisfy the need for immunity at different stages of HIV infections. Such signatures can form a task-evoked network representing HIV reservoirs at a global level of network organization. Circles in blue and magenta represent different immunologic signatures (serve as *vertices*) assigned to various tasks, thus clustering into disproportionate blocks of modules. *Edges* were calculated based on correlation coefficients between two adjacent *vertices*

Based on their topology and complexity, a wide variety of graphs can be defined, such as *simple graphs* (Fig. 1a), *directed graphs* (*digraphs*) (Fig. 1b), *disconnected graphs* (Fig. 1c), *planar graphs* (Fig. 1d), *forests/trees* (Fig. 1e and 1f), and so on. According to its name, it is easy to assert that a *simple graph* is a graph with the simplest structure. In principle, a *simple graph* consists of two elements—$V$ and $E$, where $V$ is a finite set of *vertices* (*nodes*) and $E$ is a finite set of *edges*, representing two-element subsets (e.g., $i$ and $j$) of $V$ (Fig. 1a). A *simple graph* can be written as

1 $$G=(V,E)$$

Due to text-length limits in the main text, the definition of each type of graph is summarized in Box 1 and in the *Glossary*.

### What is network topology?

The topology of a network is pivotal in determining the network properties associated with its functional annotations. It describes the way in which the *vertices* and *edges* are arranged within a network. Topological properties reflect the architecture of a network. Measuring the network topology and extracting meaningful information from the overall or particular topological properties of a network can be essential to comprehending its biological interpretation.

There are at least three key topological properties, including *small-world* [47], *scale-free* [48], and *self-similarity properties* [49]. The Erdös–Rényi (ER) model first illustrated the mathematical property of a random network, which starts with N *vertices* and connects each pair of *vertices* with probability *p*, creating a graph with approximately *p*N(N–1)/2 randomly placed links. The *vertex* degrees follow a Poisson distribution, which indicates that most *vertices* have approximately the same number of links (close to the average degree $\left\langle k \right\rangle$, where $\left\langle \ \right\rangle$ denotes the average). *Small-world properties* can be characterized by the mean *path* length, *l*, which is proportional to the logarithm of the network size, *l* ~ log N, where *l* represents the average over the shortest *paths* between all pairs of *vertices*, offering a measure of a network’s overall navigability.

Although the investigated random network model from Paul Erdös and Alfréd Rényi assumes that a fixed number of *vertices* are connected randomly to each other, reflecting the *disassortative* nature of networks, it has been shown that *small-world* networks can be highly clustered, like regular networks, yet have the characteristic of small *path* lengths, which is similar to random graphs [47]. In other words, the connection topology of *small-world* networks lies between those of regular and random networks, subsequently forming clusters (i.e., network modules).

*Disassortativity* (see below for details) seems to be a property of biological (e.g., metabolic and protein interaction) networks, such as *ultra-small-world properties*, in which the *path* length is much shorter than that predicted by the *small-world* effect. This *ultra-small-world* effect in the cell was first documented for metabolism [50], where *paths* harboring only three to four reactions can link most pairs of metabolites. Within such a network, a module usually represents a community of *vertices*, which are interconnected with each other, but are weakly connected to *vertices* in other communities [51]. The *vertices* localized in the same modules tend to perform the same, or similar, biological functions and represent a part of a signaling pathway or certain complex cellular machinery. Classic examples of biological networks in HIV and other organisms will be highlighted in *the next section*. It is still important to note that whether or not a disconnection between subnetwork graphs reflects a discrepancy in their functional annotations requires further investigation.

### Network measures

Shortest *paths*—or the shortest distance between any two *vertices*—are frequently used to model how information flows in biological networks. The degree—which indicates the number of *edges* that connect to a *vertex*, i.e., *edge* connectivity or *edge* connectedness—is one of the fundamental parameters that determines the centrality of a network and influences its network topology. The topology of a network in which the *vertices* possess a high degree of *edge* connectivity tends to be *assortative*, whereas a low degree of *edge* connectivity of *vertices* renders the topology of a network *disassortative* [52].

The concept of *assortativity* describes the correlations of *vertex* properties across edges [52]. Within a network, each *edge* is usually associated with two degrees—one for the *vertex*, where the *edge* originates, and another for the *vertex*, where the *edge* points. A possible degree correlation between the *vertices* across the *edges* illustrates the topology of a network and can be represented by its *assortativity*. *Assortativity* coefficients ranging between -1 and 1 can be used to describe the *assortativity* of a network. When the coefficient is positive (> 0), namely *assortativity* or *positive assortativity*, *vertices* tend to connect to others with similar properties within a network, whereas when the coefficient is negative (< 0), so-called *disassortativity* or *negative assortativity*, there is a propensity for *vertices* to connect to others with distinct properties within a network. In general, social networks are mostly assortatively mixed, but technological and biological networks tend to be *disassortative* [52].

Real-world networks are often heterogeneous and have few types of *vertices*, including so-called “driver nodes”*,* with well-defined topological characteristics and specific roles in controlling and driving the networks into specific states [53]. Consistently, in many biological networks, there are a few *vertices* with a disproportionately high *edge* connectivity—so-called hubs [54]. When the degree distribution of *edge* connectivity follows a power law function, the network is often referred to as being *scale-free* due to the lack of a modal hump characteristic of the Poisson distribution [48]. It is important to stress that simulation results indicate that driver nodes in both model and real systems do not necessarily correlate with the degree of connectivity [53]; in fact, the driver nodes tend to avoid high-degree *vertices* [53]. The importance of the driver nodes (or hubs) described in biological networks is determined by the number of incoming and outgoing *edges* of *vertices* and is independent of where those *edges* point. The *self-similarity property* refers to the presence of a self-repeating pattern in the network structure, i.e., an invariant degree distribution under renormalization [49].

### The possible interpretation of biological roles in a network

It is not a simple task to assign biological functions to a network, which is, in great part, due to the computational intractability of a complete comparison between graph networks, as well as a lack of sophisticated and quantitative methods to achieve this. In addition, appropriate methods for defining a measure of the distance between graphs are still missing. However, as previously mentioned, defining network modules is one of the common strategies to overlay a network with potential biological functions, whether or not centralities or *vertices* possessing abundant *edge* connectivity resemble superior biological determinants in a biological system still requires careful examination. To gain better insights into the information encoded in network topology, various methods involved in “network comparison”, allowing for the detection and interpretation of patterns of changes in networks, have been explored [55]. Seeking *graphlets* [56]—a set of driver *vertices* defined as small-induced subgraphs—is one such approach. *Graphlets* are small, connected, non-isomorphic, induced subgraphs of a network [56], which can be used to derive detailed descriptions of the network topology, unraveling the structure at the *vertex* and *edge* levels [57]. *Graphlet*-based tools have been evaluated regarding the fit of biological network models [57]. More recently, Wang et al. proposed the application of partial correlation networks—a specific kind of Markov field model—for the analysis of multi-modal datasets [58] to eliminate spurious correlations caused by unmapped biological networks and to identify critical immune interactions, particularly in settings with limited data [58]. Nevertheless, graph networks facilitate the retrieval of hidden biological information; however, the need for approaches that enable the translation of network topology into everyday language and provide a clear real-world interpretation is still imperative. In long-term perspectives, the identification of core–peripheral structures within a network in conjunction with infectious diseases may unveil variations across individuals and serve as an approach applied to personalized medicine and predictive health (see “Outstanding questions”).

## Network-based analyses of the HIV reservoir

### A functional linkage between HIV and the properties of the host functional genome

HIV pathogenesis is strongly associated with the host genome, as only integrated proviruses can achieve persistent infections. The selection of HIV integration sites is biased through the chromosomes [59–61]. At the genetic level, around 40% of the integrations are in clonally expanded cells, and multiple independent integrations favor cancer-related genes [62]. A study conducted by Zhyvoloup et al. [63] suggested a functional link between HIV integration preference and T cell activation coupled with cell metabolism. Several metabolic pathways associated with the active replication of retroviruses and the entry of their latency have been explored and investigated in the pursuit of a functional cure for the eradication of HIV. One of the first metabolic pathways that was explored is nucleotide metabolism. From this root of metabolic pathways, several others have also been identified, e.g., cells with increased glycolytic rates are susceptible to HIV infections [64]. Shytaj et al. further demonstrated that the entry of HIV latency is accompanied by the downregulation of glycolysis, coupled with a higher reliance on the antioxidant thioredoxin and glutathione systems for cell survival [65]. The molecular basis of antioxidant pathways involved in the development of HIV latency has been further clarified, in that the protein turnover of promyelocytic leukemia protein nuclear bodies, resulting from post-translational modifications (i.e., SUMOylation and ubiquitination), determines the entry and exit of latency in proviruses [66]. Although these studies have made remarkable progress in reinforcing the concept of a functional linkage between HIV pathogenesis and cellular functionality, the ways in which the intricate realities of cellular pathways affect HIV pathogenesis require further investigation. In this context, network-based analyses enable investigations from a global level, regarding the network organization between these two counterparts.

### Network-based analyses unravel interactions between HIV and cellular functionality

Network-based analyses have been applied to a broad variety of omics studies related to HIV, utilizing epigenomics, genomics, transcriptomics, proteomics, and interactomics data. One strategy to converge omics datasets from both the host and HIV aligns with the principle of *bipartite graphs*, representing a graph in which the *vertex* set can be split into two disjoint subsets, as well as each *edge* of a graph joining a *vertex* of the two subsets (Fig. 1h). Based on this principle, MacPherson et al. developed a methodology called biclustering, which allows for a connection between the readouts from the Human Protein Interaction Database and those from global siRNA screening for the identification of host factors required for HIV replication [67]. The same methodology and principle have also been applied to investigate the interactions between HIV and human proteins at the higher level of network organization.

In order to better characterize the microenvironment of HIV reservoirs, our laboratory recently proposed the hypothesis that HIV reservoirs can be represented by the task-evoked property of a topological network consisting of different communities of host genes being targeted by HIV (Fig. 1i) [1–3]. Such communities are so-called “immunologic signatures” [1, 3]. The HIV integration frequency within a network might be used as a surrogate for immunologic signatures in order to define specific immune cell types and proinflammatory soluble factors, subsequently satisfying the need for host immunity alongside HIV infections [1, 2]. A comparison between immunological features with the potential to serve as biomarkers was determined using the network-based analysis; those features identified based on longitudinal clinical samples (Table 1) will be summarized and discussed in the following section.

Building on this hypothesis, a network was constructed using HIV-targeted genes as entities (Fig. 1i), followed by immunologic signatures (gene sets) and a task-evoked network in a hierarchical order [3]. Differing from the previously mentioned strategy, our approach directly utilizes the host genes that present physical contact with HIV (HIV DNA integration) to construct a network. In our setting, a *vertex* represents an immunologic signature. We retrieved predictor variables and assigned them to three categories, including (1) variables related to immunologic signatures, (2) host gene expression, and (3) variables associated with the 3D genome. Different combinations of the predictor variables were used to measure correlation coefficients, representing *edges* between two adjacent *vertices*, thereby creating a network topology [3]. We observed that the implementation of different categories of predictor variables led to distinct topological structures of the networks; in particular, the variables related to immunologic signatures offer a better classification of the network topology in reservoirs between ART-treated patients and elite controllers [3]. See Box 2, which highlights the potential interplay between elite controllers and the host genome.

While annotating biological functions on networks at the level of immunologic signatures, it was observed that distinct surface markers are associated with enriched signatures in ART-treated patients and elite controllers [3]; additionally, at the network level, a high frequency of enriched signatures possessing the highest *edge* connectivity was present in ART-treated patients, which was associated with regulatory and conventional T cells and CD4 thymocytes, whereas signatures possessing the lowest *edge* connectivity were mainly present in elite controllers and were associated with CD8 T cells and B cells [3]. To some extent, this observation verified the mentioned hypothesis that distinct functional annotations related to immunity were assigned to distinct networks, representing HIV reservoirs between ART-treated patients and elite controllers. Such a correlation between the connectedness of *vertices* and immunity still requires clinical verification. Another critical issue is how to robustly assign biological functions to a network (see “Outstanding questions”).

### A possible intra-host evolution of the configuration of HIV reservoirs

Markov chain modeling analyses have been widely applied in studies on HIV, with emphasis being placed on epidemiological investigations. The studies of Mathieu et al. [68], Binquet et al. [69], and Shoko and Chikobvu [70] involved using the continuous-time Markov model constructed with clinical features, including the CD4 T cell count [68], viral load measurement [68], parameters associated with disease progression and immunological deterioration [69], and tuberculosis co-infection [69], allowing for the subsequent identification of potential predictor variables for HIV/acquired immunodeficiency syndrome (AIDS) progression in patients subjected to ART and immune deterioration. Wan et al. applied the Ornstein–Uhlenbeck model, which is a temporally homogeneous Markov model, to investigate the heritability of the HIV reservoir size, as well as the decay of the reservoir under long-term suppressive ART [71].

**Fig. 2:**
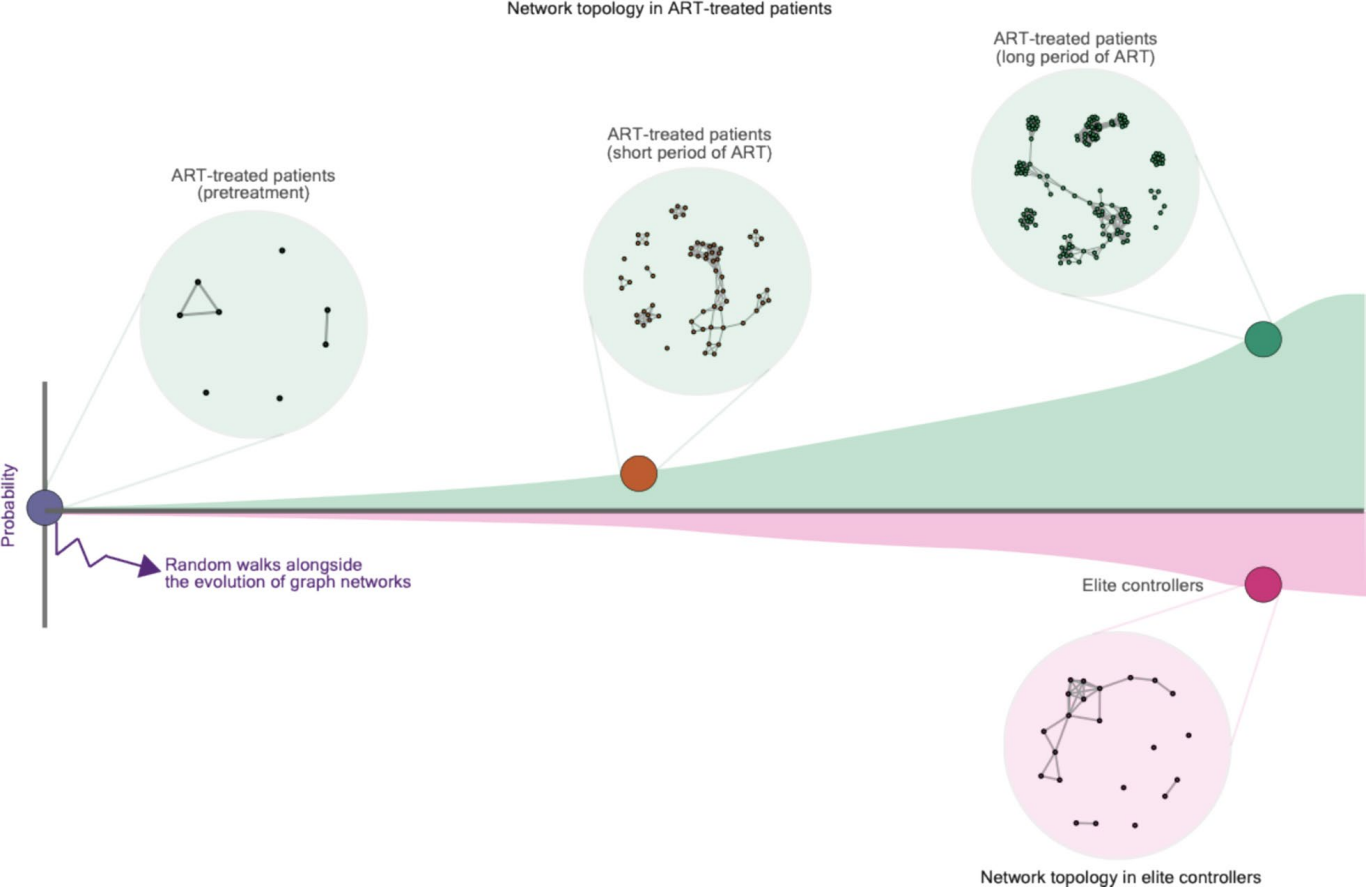
Schematic representation of the evolution of the graph network, representing the configuration of HIV reservoirs alongside HIV infections associated with ART and that structured in elite controllers. As computing the Pearson distance between the graph networks from reservoirs in pretreatment HIV-infected individuals, patients subjected to short and long periods of ART (graph networks highlighted in green circles), and elite controllers (the graph network highlighted in a pink circle), it is observed that the graph network between patients subjected to a long period of ART and elite controllers demonstrated the farthest graph distance, suggesting that a lack in the graph isomorphism of the networks between these two types of HIV-infected individuals. More details can be referred to as Wiśniewski et al. [3]

In our study, we also attempted to tackle the question of whether the graph network that represents HIV reservoirs evolves and responds to different stages of HIV infections [3]. If it is true, does the evolutionary progression of a graph network in ART-treated patients (non-controllers) differ from that in elite controllers (Fig. 2)? We constructed a task-evoked property of a network that consists of different communities (i.e., immunologic signatures) of host genes targeted by HIV and used it to represent HIV reservoirs appearing at different stages of HIV infections associated with ART and elite controllers [3]. First, a limited number of immunologic signatures were shared across networks constructed at different stages of HIV infections associated with ART and with those enriched in elite controllers [1, 3], indicating that enriched signatures are most likely transient and unique to particular phases throughout the progression of HIV infection. Furthermore, we illustrated the evolution of a graph network using longitudinal samples from HIV-infected individuals, where *edges* were calculated based on the Pearson correlation coefficients between two adjacent *vertices*. We observed a lack of graph isomorphism between the networks of ART-treated patients in a longitudinal order and elite controllers [3] (Fig. 2), implying that distinct core–peripheral structures (e.g., *graphlets* [56]) are present between elite controllers and ART-treated patients. It is also important to note that at present, the interaction between consistent and emerging signatures alongside the network evolution of HIV reservoirs is not fully understood. Further profound investigation is imperative to dissect their topological interactions and functional discrepancies.

In addition, we simulated random walks across graph networks in ART-treated patients in a longitudinal order and elite controllers using Markov chain Monte Carlo modeling, coming to the conclusion that an intrinsic barrier of the network topology lies between non-controllers and elite controllers. In particular, a transition in the graph network between elite controllers and non-controllers should be rare in the real world. It is important to note that a noticeable probability of the transition between the graph networks from elite controllers to ART-treated patients subjected to a long period of ART [3] may reflect the clinical observation that elite controllers may experience occasional viral load ‘‘blips’’ above the level of detectability when using conventional assays [72–74].

## Potential longitudinal biomarkers targeting the HIV reservoir

### Search strategy and selection criteria

A rigorous literature search was conducted using PubMed with the keywords ((longitudinal) AND ((HIV reservoir) OR (latent HIV reservoir) OR (HIV latency))) (accessed on June 2024). It is acknowledged that the literature is very abundant and to avoid the topics that have been frequently discussed, I thus focused on research articles from the last five years (2019–2024), with the limitation of solely selecting research articles published in the English language, with the exclusion of review articles and preprints. After careful examination, 44 articles were included, in which longitudinal research was conducted fitting the scope of this review; these are summarized in Table 1. Apart from these collected articles, Borgognone and colleagues have reported that gut microbiome and host immune-activation signatures are inversely correlated with HIV reservoir size [75]. Gianella and colleagues have demonstrated that clinical characteristics, i.e., age and sex, also impact the rate of total HIV DNA decrease over time across patients [76]. Peripheral proviral DNA, which was identified in simian immunodeficiency virus-infected rhesus macaques has been suggested to be a surrogate for the estimation of HIV reservoir size, as well as a predictor variable for viral rebound after treatment interruption [77]. Given that few studies with respect to these biomarkers can be found, in addition to text-length limits, such biomarkers will not be discussed in the following content.

### Longitudinal biomarkers related to HIV

The measure of HIV genetic materials (DNA and RNA) is one of the straightforward indicators that is used to estimate the size of the HIV reservoir. Among the 14 articles [4–17] in this category, three of them investigated the HIV reservoir in the central nervous system [9, 17], and two of the studies were based on the reservoir in infants [14, 16]. Sharma and colleagues (2021) demonstrated that spliced HIV RNA appears in the cerebrospinal fluid of CD4 T cells during the earliest stages of infection in simian-human immunodeficiency virus (SHIV)-infected rhesus macaques, resembling the observation in human acute HIV infection, whereby peak plasma SHIV RNA occurs two weeks post-infection, followed by a gentle decrease [9]. DeMarino and colleagues (2024) also detected HIV RNA in extracellular vesicles derived from cerebrospinal fluid and serum, observing a negative correlation between the copy number of the TAR region, marking the transcription initiation/elongation block in the R region of 5’ long terminal repeat (LTR) and the overall neurocognitive function [17].

Other studies of longitudinal biomarkers were conducted with HIV-infected CD4 T cells and peripheral blood mononuclear cells (PBMCs) from the blood, representing the main reservoir cells of HIV. Of note, a study from Cannon et al. [15] utilized near full-length HIV sequences coupled with a machine-learning approach and mathematical models. The measure of HIV genetic materials [7, 10, 13] and the level of viremia [4] can be used to indicate the progressive development of the reservoir in the presence of the treatment or after the treatment is interrupted. The amount of HIV DNA and RNA was reported to positively correlate with immune reconstitution after the treatment was given [13]. Viral blips and a low level of viremia were significantly associated with slower reservoir decay [4]. It is important to note that HIV RNA splice donor site D1 [15] and the ratio between unspliced and multiply spliced RNA from HIV associated with cells [8, 15] were observed to be associated with the reservoir dynamics and better immune reconstitution, respectively. In addition, the integrity of the HIV genome has also been taken into account, whereby intact HIV DNA, which rapidly decays in circulating CD4 T cells [12], is associated with residual plasma viremia [6] and immunosuppression [16], whereas defective proviruses with solo LTR can persist in infected cellular clones in infants on ART [14]. However, HIV 2-LTR circles demonstrate a non-linear decay compared with intact proviruses [12]. As a final remark in this section, the HIV *nef* and *tat* genes, the sequence of the *env* gene, and mutations identified in HIV reverse transcriptase were also reported for the surveillance of the latent reservoir [15] and the clonal expansion of the reservoir cells [15]. Additionally, viral kinetics between the upper and lower genitourinary tract [5], as well as viral haplotypes in conjunction with antiretroviral drug resistance in the reservoir [11], were also reported.

### Longitudinal biomarkers related to immune cells

**Fig. 3:**
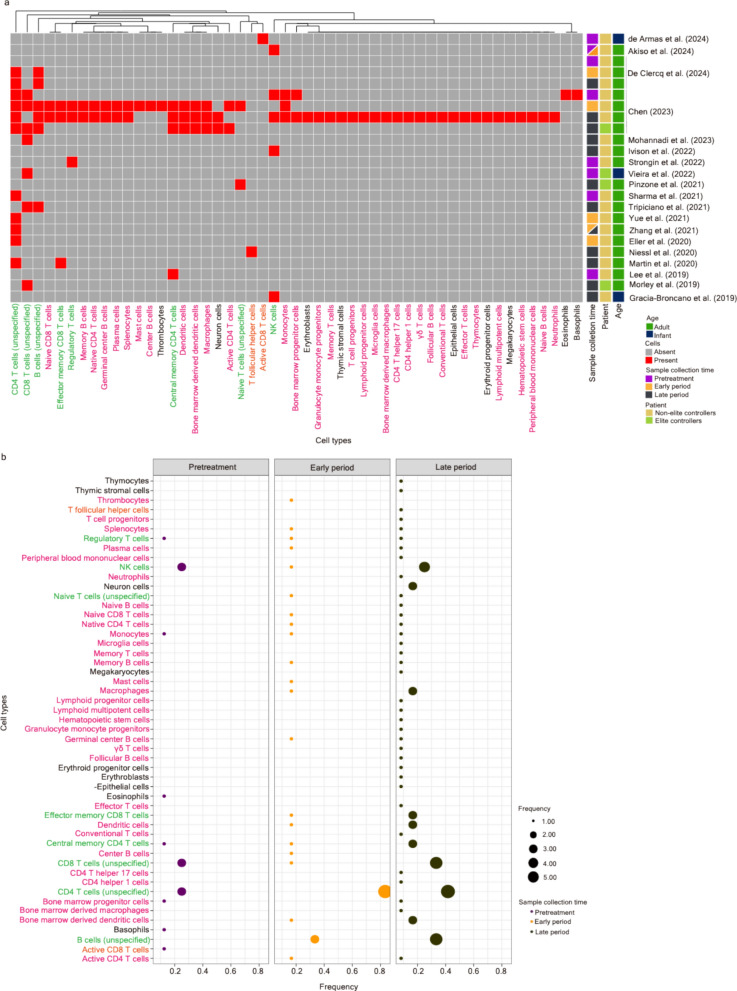
A systematic comparison of the potential longitudinal biomarkers associated with various cell types across the literature. **a** A clustering heatmap displaying the appearance of cell types (including immune cells and other cell types shown in the column) across 19 research articles. Immune cell types identified based on experimental studies using longitudinal clinical samples are marked in orange; immune cell types identified based on network-based analysis are marked in magenta; immune cell types unveiled in both scenarios are written in green. Squares marked in red indicate the appearance of corresponding cell types; opposites are marked in grey. Heatmap annotations from the left-hand side include (1) Sample collection time—samples collected in the pretreatment period (including the phase of acute HIV infection; written as “pretreatment”) are marked in dark purple, samples collected within one year after the initiation of ART (written as the “early period”) are marked in orange, and samples collected more than one year after the initiation of ART (written as the “late period”) are marked in dark green; (2) Patient—samples collected from ART-treated patients and non-elite controllers are marked in yellow and those from elite controllers are marked in light green; (3) Age—samples collected from adults are marked in green and those collected from infants are marked in dark blue. **b** A bobble plot representing the frequency of the appearance of cell types identified in three stages (pretreatment, early period, and late period) of HIV infections associated with ART. The frequency was calculated using the time of the appearance of cell types in each infection stage divided by the number of articles collected in the same infection stage. Color codes for immune cell types align with the description written in (**a**)

In total, 19 articles [1, 9, 10, 18–22, 25–30, 32, 33, 35, 36, 42] have demonstrated the appearance of various types of cells across different stages of HIV infections and disease progression based on experimental research using longitudinal clinical samples and network-based analyses, as summarized in Table 1 and Fig. 3a. Based on the sample collection time aligning with the HIV/AIDS disease progression and the course of ART treatment, three main blocks of infection periods associated with ART have been designated—(1) pretreatment (including acute HIV infection), (2) less than one year after the initiation of ART (hereinafter referred to as the “early period”), and (3) more than one year after the initiation of ART (hereinafter referred to as the “late period”) (Fig. 3a and b). A propensity that an increasing variety of cell types reported at the late period was observed (Fig. 3a).

Within immune cells, active CD8 T cells [42] (pretreatment) and T follicular helper cells [22] (late period) were reported exclusively based on experimental research (Fig. 3a; cell names written in orange). B cells (unspecified) [1, 25, 33], CD4 T cells (unspecified) [1, 9, 10, 21, 26, 33, 35], CD8 T cells (unspecified) [1, 20, 25, 29, 30], central memory CD4 T cells [1, 19], effector memory CD8 T cells [1, 35], regulatory T cells [1, 28], naive T cells (unspecified) [1, 36], and NK cells [1, 18, 27, 32] were identified based on both experimental research and network-based analysis (Fig. 3a; cell names written in green). Immune cells written in magenta are those exclusively identified based on the network-based analysis [1].

Both active and naive T cells [1, 9, 10, 21, 26, 33, 35, 36] and their subsets, including regulatory T cells [1, 28], (central) memory CD4 T cells [1, 19], and CD8 T cells [1, 20, 25, 29, 30], have been reported across the three periods; other subsets, including conventional T cells [1]; effector T cells, including Th1 and Th17 [1]; effector memory CD8 T cells [1, 35]; γδ T cells [1], and T follicular helper cells [22], preferentially appear after the initiation of ART (Fig. 3a). Indeed, as the main reservoir cells of HIV infection, the dynamic of the absolute CD4 T cell count has been used as one of the important indices for the evaluation of disease progression and immune restoration after ART, whereas CD8 T cells function to eliminate infected cells (e.g., the HIV-specific CD8 T cell response; see Box 2). The study of de Armas et al. [42] demonstrated the presence of a subset of CD8 T cells, which display gene profiles that are consistent with cytotoxic T lymphocytes in HIV-exposed infected infants, likely influencing the establishment of the HIV reservoir. Of note, the appearance of CD8 T cells (unspecified) showed a higher frequency than other immune cell types in elite controllers [1, 20, 29] (Fig. 3a; see Box 2).

Many B cell defects in HIV infection are associated with alterations in the B cell subpopulations that circulate in the peripheral blood. Several minor subpopulations of B cells, including immature transitional B cells, exhausted B cells, activated mature B cells, and plasmablasts have been reported to be over-represented in HIV-infected individuals [78]. Based on selected longitudinal studies, the majority of B cells appear after the initiation of ART [1, 25, 33] (Fig. 3a). Other immune cells, particularly infected macrophages and microglia cells, may also contribute to persistent HIV infections, at least in a subset of people living with HIV. Although the biology of such infected cells is not well characterized, a number of studies have identified their dynamic involvements in the course of HIV infection.

Dendritic cells (DCs) and bone marrow-derived DCs appear after the initiation of ART [1] (Fig. 3a). Although the study from Martyn-Gayo and colleagues stresses the importance of DCs in controlling viral replication in elite controllers [79], the numbers of DCs diminished during the period of pretreatment [80, 81]. It is important to stress that recent studies utilising single-cell RNA sequencing have redefined the repertoire of DCs in blood—AXL^+^Siglec-6^+^- [82] and AXL^+^Siglec-1^+^ [83] DCs, which may be involved in HIV transmission and the spread at the very early stage of HIV infection in patients.

Natural killer (NK) cells also appear across three periods [1, 18, 27, 32]. It has been reported that the number of functional NK cells (CD56^dim−^CD16^+^ subset), which present the majority of NK cells at the acute phase of HIV infection, shows an increase and then a decrease in the chronic phase of infection [84, 85]. Although it has been suggested that ART can help recover NK cells [84], whether NK cell activation is associated with HIV disease progression is presently debated. A recent study has reported that higher frequencies of functional CD56^bright^ NK cells with increased cytotoxic activity are detectable in nine young adults who acquired HIV perinatally and remained on suppressive long-term ART (median: 20 years) since infancy [86]. However, another clinical study suggested that early-life HIV/ART exposure may not result in major changes in NK cell subsets in 5-year-old children [87].

Overall, the frequency of immune cells that could serve as potential longitudinal biomarkers across three periods is summarized in Fig. 3b. Among all types of immune cells discussed in this section, CD4 T cells (unspecified) are frequently chosen as the potential biomarker across all periods (Fig. 3b). However, it is important to note that some types of immune cells that are observed across more than one period, according to different studies, might be due to either different strategies of sample acquisition or different analytical methods. In the future, a more precise window of time relating to HIV infections associated with ART will be required for the better characterization of longitudinal biomarkers.

### Longitudinal biomarkers related to cellular molecules and soluble factors

**Fig. 4:**
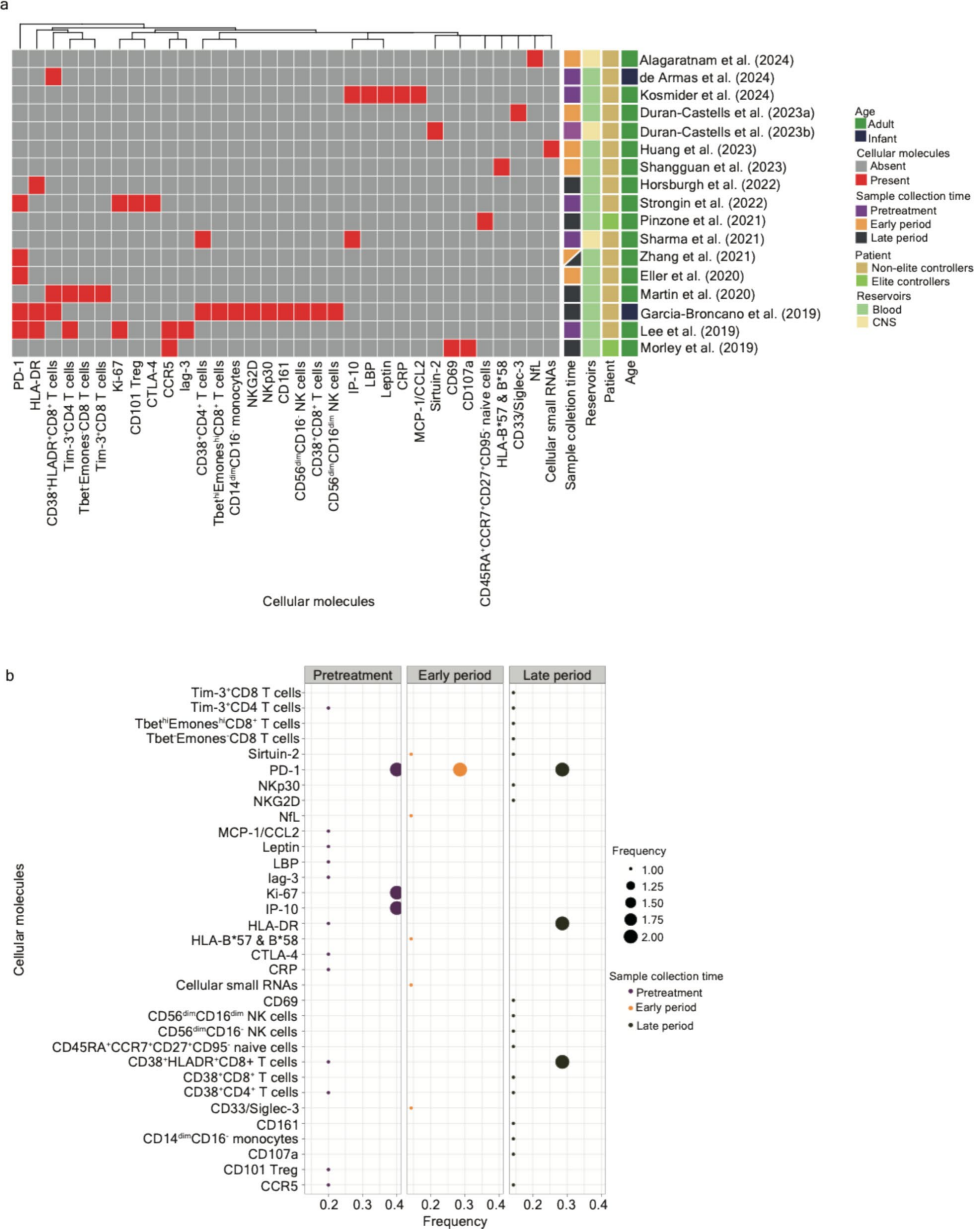
A systematic comparison of the potential longitudinal biomarkers associated with cellular molecules and soluble factors across the literature. **a** A clustering heatmap displaying the appearance of cellular molecules and soluble factors (column) across 17 research articles. Squares marked in red indicate the appearance of corresponding cellular molecules and soluble factors; opposites are marked in grey. Heatmap annotations from the left-hand side include (1) Sample collection time—samples collected in the pretreatment period (including the phase of acute HIV infection; written as “pretreatment”) are marked in dark purple, samples collected within one year after the initiation of ART (written as the “early period”) are marked in orange, and samples collected more than one year after the initiation of ART (written as the “late period”) are marked in dark green; (2) Reservoirs—samples collected from blood are marked in light green and those from the materials in the central nervous system are marked in light yellow; (3) Patient—samples collected from ART-treated patients and non-elite controllers are marked in yellow and those from elite controllers are marked in light green; (4) Age—samples collected from adults are marked in green and those collected from infants are marked in dark blue. **b** A bobble plot representing the frequency of the appearance of cellular molecules and soluble factors identified in three stages (pretreatment, early period, and late period) of HIV infections associated with ART. The frequency was calculated using the time of the appearance of cellular molecules and soluble factors in each infection stage divided by the number of articles collected in the same infection stage

C–C chemokine receptor type 5 (CCR5) [19, 20], CD33/Siglec-3 [38], CD69 [20], CD107a [20], CD101 (marked on regulatory T cells) [28], CD161 [18], C-reactive protein (CRP) [34], cytotoxic T-lymphocyte associated protein 4 (CTLA-4) [28], human leukocyte antigen allele HLA-B*57 and B*58 [41], human leukocyte antigen-antigen D related (HLA-DR) [18, 19, 35, 37, 42], interferon gamma-induced protein 10 (IP-10) [9, 34], Ki-67 [19, 28], lymphocyte-activation gene 3 (Lag-3) [19], lipopolysaccharide binding protein (LBP) [34], leptin [34], monocyte chemoattractant protein-1 (MCP-1/CCL2) [34], neurofilament light protein (NfL) [31], NKG2D (NK cell receptors) [18], NKp30 (NK cell receptors) [18], programmed cell death protein 1 (PD-1) [18, 19, 21, 26, 28], sirtuin-2 [39], T-bet (T-box expressed in T cells) and Eomesodermin (Emones) [18, 35], T cell immunoglobulin and mucin containing protein-3 (Tim-3) [19, 35], and cellular small RNAs [40] have been reported to be significant during different stages of HIV infections (Table 1 and Fig. 4a).

Molecules, including CD14^dim^CD16^−^ monocytes; CD38^+^CD8 T cells; CD56^dim^CD16^dim^ and CD56^dim^CD16^−^ NK cells; and CD161, NKG2D, NKp30, and T-bet^hi^Emones^hi^CD8 T cells, were reported only in neonates with HIV infections [18], whereas CD38^+^CD4 T cells [9, 18], HLA-DR [18, 19, 35, 37, 42], and PD-1 [18, 19, 21, 26, 28] were identified in both neonates and adults.

Several factors unveil their significance for HIV infection within the central nervous system (CNS). CD38 expression increased in both CD4^+^ T cells in the cerebrospinal fluid four weeks post-infection, and the chemoattractant IP-10 was elevated at the eighth week [9]. Sirtuin-2 [39], an NAD-dependent deacetylase, was reported to be strongly associated with elevated viral loads, HIV provirus levels, and markers of neurological damage, including NfL [31].

Using the simian immunodeficiency virus (SIV) model of HIV infection in rhesus macaques, Strongin and colleagues observed the depletion of CD101^+^CD4 T cells during acute SIV infection [28]; the reconstitution of CD101^+^CD4 T cells accompanies highly expressed PD-1 and CTLA-4 levels [28], both representing an essential cellular intrinsic mechanism that controls overt immune responses to maintain immunological homeostasis, and a high level of Ki-67 [19, 28], which is a cell proliferation index. It is important to note that PD-1 has also been observed as a phenotypic signature, marking HIV reservoir cells harboring intact proviruses [88].

Previous studies have demonstrated the association between CD4 memory T cells with HLA-DR expression and the persistence of integrated HIV DNA and disease progression [89, 90]. Lee and colleagues observed that CD4 memory T cells that express HLA-DR were detectable in the stage of acute HIV infection [19]; the average expression levels of CCR5, PD-1, and Tim-3 were higher in the HLA-DR^+^ T cell subset, whereas the average Lag-3 expression was higher in their HLA-DR^−^ counterparts [19]. Lag-3 and Tim-3 are markers for immune exhaustion [91]. CCR5 is critical for virus-mediated pathogenesis and T cell co-stimulation [92, 93]. CD38 is another activation marker of T lymphocytes. As previously mentioned, de Armas and colleagues have reported that CD38^+^HLA-DR^+^CD8 T cells correlate with viral load in perinatal HIV [42].

Other molecules and factors include CRP, which is also a biomarker of inflammation coupled with infections [34]. In addition, a decrease in leptin, which is a marker of energy expenditure, from pretreatment to HIV post-infection has been observed in ART-treated patients [94]. The same pattern was also found in LBP, which showed a decrease immediately after ART treatment [34]. The plasma level of CD33/Siglec-3 was reported to be a biological marker of HIV control in the absence of ART [38]. B*57:01 and B*58:01 were associated with the reduced size of the HIV reservoir over time in people living with HIV on suppressive ART [36, 41]. It is important to stress that CD69- [20], CD107a- [20], and CD45RA^+^CCR7^+^CD27^+^CD95^−^-naive cells [36] that were present at an extremely low level were reported in elite controllers (Table 1, Fig. 4b, and Box 2). To summarize, PD-1 is the factor that is frequently reported across three periods alongside HIV infections, whereas Ki-67 [19, 28] and IP-10 [9, 34] are frequently reported in pretreatment HIV-infected individuals, and HLA-DR [18, 19, 35, 37, 42] and CD38^+^HLADR^+^CD8 T cells [18, 35, 42] are frequently reported in patients subjected to a long period of ART.

### Longitudinal biomarkers related to proinflammatory factors

**Fig. 5:**
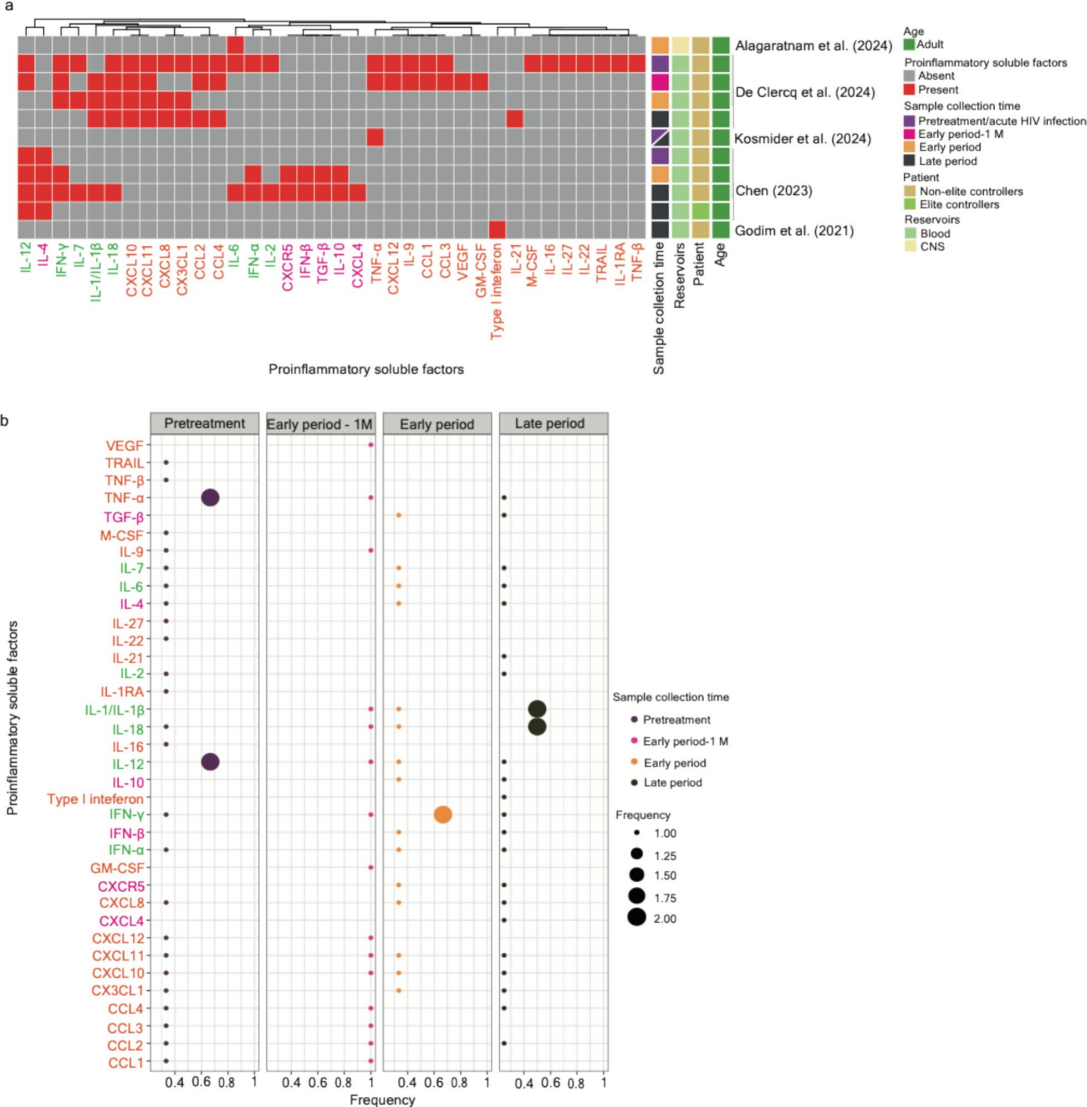
A systematic comparison of the potential longitudinal biomarkers associated with proinflammatory factors across the literature. **a** A clustering heatmap displaying the appearance of proinflammatory factors (column) across 5 research articles. Proinflammatory factors identified based on experimental studies using longitudinal clinical samples are marked in orange; proinflammatory factors identified based on network-based analysis are marked in magenta; proinflammatory factors unveiled in both scenarios are written in green. Squares marked in red indicate the appearance of corresponding cell types; opposites are marked in grey. Heatmap annotations from the left-hand side include (1) Sample collection time—samples collected in the pretreatment period (including the phase of acute HIV infection; written as “pretreatment”) are marked in dark purple, samples collected about one month after the initiation of ART (written as the “early period-1 M”) are marked in magenta; samples collected within one year after the initiation of ART (written as the “early period”) are marked in orange, and samples collected more than one year after the initiation of ART (written as the “late period”) are marked in dark green; (2) Reservoirs—samples collected from blood are marked in light green and those from the materials in the central nervous system are marked in light yellow; (3) Patient—samples collected from ART-treated patients and non-elite controllers are marked in yellow and those from elite controllers are marked in light green; (4) Age—samples collected from adults are marked in green and those collected from infants are marked in dark blue. **b** A bobble plot representing the frequency of the appearance of cell types identified in three stages (pretreatment, early period-1 M, early period, and late period) of HIV infections associated with ART. The frequency was calculated using the time of the appearance of proinflammatory factors in each infection stage divided by the number of articles collected in the same infection stage. Color codes for immune cell types align with the description written in (**a**)

A total of 36 proinflammatory factors unveiled their significance at different stages alongside HIV infections associated with ART (Table 1 and Fig. 5a). Among them, 22 factors were identified based on experimental research with longitudinal clinical samples—interleukin-1 receptor antagonist (IL-1RA), IL-16, IL-22, IL-27, macrophage colony-stimulating factor (M-CSF), tumor necrosis factor-β (TNF-β), and TNF-related apoptosis-inducing ligand (TRAIL) were observed in the stage of acute HIV infection [33]; type-1 interferon was observed in pretreatment HIV-infected individuals [23]; granulocyte–macrophage colony-stimulating factor (GM-CSF) and vascular endothelial growth factor (VEGF) were observed in patients subjected to ART within a month [33]; IL-4 was observed in the late period; CCL1, CCL3, CXCL12, IL-9, and TNF-α showed an enrichment in the stage of acute HIV infection and patients subjected ART (less than 1 month); and the expression of CCL2, CCL4, CXCL10, CX3CL1, CXCL8, and CXCL11 was significant across more than two different stages or the whole course [33] (Fig. 5a). Of note, in another study, TNF-α was also reported to be observed in patients subjected to a long period of ART [34].

Six factors were retrieved using network-based analysis (Table 1 and Fig. 5a), as follows: CXCL4 was observed in patients subjected to a long period of ART [1]; CXCR5, IL-10, interferon-β (IFN-β), and transforming growth factor-β (TGF-β) were observed in patients subjected to a short and long period of ART [5]; and IL-4 was observed across all stages [1]. Although eight factors, including IL-1/IL-1β, IL-2, IL-6, IL-7, IL-12, IL-18, IFN-α, and IFN-γ, were unveiled based on both experimental investigation and the network-based analysis, a great discrepancy in the appearance of potential longitudinal biomarkers was observed [1, 33]. A further investigation with more precise windows of time alongside the course of HIV infection will be required. Of note, IL-6 was reported in the CNS (early period) [31], while IL-4 and IL-12 were observed in elite controllers (late period) [1]. It is worth noting that Lee and colleagues using RNA sequencing datasets subjected to network-based analysis observed that gene sets involved in the TGF-β signaling pathway showed an enrichment in long-term non-progressors compared to healthy donors. However, a downregulation of the expression of genes involved in the IFN-α response was observed [95] (Table 1). In this section, I acknowledge that the literature with respect to the function and the consequence of the mentioned proinflammatory factors on HIV is very abundant and has been previously well discussed; thus, I cannot cover it exhaustively nor do I want to repeat frequently discussed topics. Overall, IL-12 and TNF-α were frequently shown in acute HIV infection and pretreatment HIV-infected individuals, IFN-γ was frequently present in the early period, and IL-1/IL-1β, IL-4, IL-12, and IL-18 were frequently observed in the late period (Fig. 5b).

### Longitudinal biomarkers related to the host genome, host factors, and epigenomes

Although the fundamental mechanism of how the host genome is involved in determining HIV pathogenesis remains not fully understood, evidence supported by our previous study [96] and many others [97–100] has underscored the local genomic context coupled with epigenetic features in the regulation of stochastic HIV transcription and reactivation, perhaps subsequently regulating the entry and relief from HIV latency. The same finding was observed using longitudinal samples harboring HIV infections, where the detectable integration sites of producer proviruses that correspond to plasma HIV RNA sequences were enriched in proximity to the activating H3K36me3 epigenetic mark [30] (Table 1). Studies from elite controllers [101] and the proposed immune-mediated selection on the configuration of the HIV reservoir [44, 102, 103] further strengthen the potential contribution of genomic locations associated with heterochromatin features (e.g., centromeric satellite DNA or zinc-finger genes) to deep latency. In addition, the pre-existing state of CpG methylation throughout chromosomes has been proposed to determine the dynamics of HIV viral rebounds and to be related to the reservoir size [46] (Table 1). The dynamics of CpG methylation on the HIV 5’LTR were also reported, whereby proviruses harboring higher levels of CpG methylation on 5’LTR tended to be present in younger patients with higher CD4 T cell counts and a dramatic decrease in CD8 T cells, resulting in greater variations in the ratio between CD4 and CD8 T cells 48 months post ART [45] (Table 1). Finally, it is known that the expression of some of the cellular proteins, namely “restriction factors”, that enable the limitation of viral replication across different steps of the viral cycle varies alongside HIV infections associated with ART. The expression of the apolipoprotein B mRNA editing enzyme, catalytic subunit 3G (APOBEC3G), bone marrow stromal antigen 2 (BST2), interferon-γ inducible factor 16 (IFI16), interferon-induced GTP-binding protein (MX2), tripartite motif-containing protein 5, α isoform (TRIM5α), SAM and HD domain-containing deoxynucleoside triphosphate triphosphohydrolase 1 (SAMHD1), serine incorporator 3 (SERINC3), and serine incorporator 5 (SERINC5) were more abundant during the diagnostic period, followed by a significant decrease after the initiation of ART [43] (Table 1).

### Outstanding questions

#### How to enhance network robustness to assign the potential functional roles of entities?

Many biological networks, especially those within the cell, approximate a *scale-free* topology, as first evidenced by the analysis of metabolism [50], thus unraveling the *disassortative* nature of biological or cellular networks. With such a *disassortative* property, hubs are formed by *vertices* possessing a high degree of connectivity [48]. Whether the presence of *vertices* in the same hubs with a high degree of functional similarity or clusters results from the probability that *vertices* prefer to connect to others that are already well-connected (i.e., so-called *preferential attachment* [48]) requires better characterization. On the other hand, an example focused on protein interaction networks indicated that *vertices* and hubs tend to connect to other proteins with little connectedness [104]. Whether such a discrepancy reflects the intrinsic differences across biological networks or a consequence of the network’s hierarchical *modularity* remains unexplained. In addition, recent studies often incorporate a broad variety of omics datasets, which may result in a loss of information or may generate boundaries between modules—such as the application of *bipartite graphs* to independent datasets—perhaps rendering existing clustering methods inefficient. At present, interpretation accuracy is challenging to measure due to the lack of appropriate gold standards [105]. The updating of conceptual frameworks and interpretation approaches to characterize the topologies of different biological networks should also be considered in the future [105, 106].

#### Is the functional property of biological networks inheritable?

Networks can be characterized as either static or dynamic entities. Static networks, as previously described, are constructed based on a sample space (i.e., a set of the possible networks $G$ defined by $V$ and $E$) [see Eq. (1)] in the “What is a network?” Section], whereas dynamic networks gather information over time and are often based on the construction of stochastic processes in the space of possible networks. In the setting of evolving networks with time, the (discrete) time variable can be defined in terms of at least two options—(1) define time instants where the probability space (i.e., the filtration $F$) does change, and (2) define only time instants where the value of the state vector changes [107]. 

*Markovian stochastic processes* (Eq. 2) are frequently implemented to model network evolution.

2 $${\{G}_{t},t\in T\}$$

Among these processes, a special class of stochastic dynamic system can be written [107], as follows:

3 $${G}_{t+1}=F({G}_{t},{w}_{t})$$

where the stochastic (random) process ${w}_{t}$ has an associated measurable space $(W,w)$ adapted to the filtration $F$.

It is important to note that *Markovian stochastic processes* can possess a discrete or continuous set of times, and the state dynamics are “memoryless”, i.e., they do not depend on how the system has led to the current state, and the state of the process at a given time contains all the information about the past evolution of the process, which is of use in predicting its future behavior. In fact, networks coupled with dynamical systems have been used to model many biological systems.

## Discussion

In this review, the advantages of applying a network-based approach, including the definition of a task-evoked property of HIV reservoirs and the identification of potential longitudinal biomarkers targeting the microenvironment of HIV reservoirs, have been highlighted. In addition to the importance of core–peripheral structures (e.g., *graphlets* [56]) of networks, which was emphasized for a better definition of the functionality of biological networks, advanced network-based technologies will also have to take efficiency, the integrity of input datasets, speed, reliability, and explainability into account. It is important to stress that although the origin of *disassortativity* in biological networks remains unexplained at present, a study based on the metabolic network of a parasitic bacterium has suggested the presence of evolutionary mechanisms to maintain the average *path* length during evolution [50]. Prediction based on a *scale-free* model also demonstrated that *vertices* that appear early in the history of the network tend to be the most-connected ones [48]. Indeed, topology alone may not be sufficient for understanding complex networks. These observations underscore the influence of temporal states on the network topology. A better understanding of how to correlate the topological alterations of an evolving network with the dynamics of biological states (e.g., cell cycles, disease progression, and the spread of diseases) and identify the emergence of temporal and *scale-free* features will allow us to gain deeper insights into the functionality of biological networks beyond their topology. Our studies based on the simulation of random walks across networks of HIV reservoirs at different stages of HIV infections associated with ART and the network representing the reservoir of elite controllers also suggested the existence of distinct evolutionary trajectories between these two types of HIV-infected individuals [3]. Presently available (longitudinal) data from elite controllers remain limited and may introduce potential biases due to data imbalance. A prudent experimental validation of the performance accuracy of outcomes from network-based analyses will be necessary to strengthen computational results.

Although network biology has been around for two decades and witnessed rapid evolution, this field remains nascent with emerging challenges. Given that network biology is an interdisciplinary domain that bridges computational and biological sciences, several critical topics, such as differential network analysis, higher-order network analysis, multimodal data integration in heterogeneous networks, and machine learning on networks, cannot be detailed in the review. Nevertheless, a closer breakdown of networks into sets of functional modules (i.e., graphlets and network motifs) and the identification of core topological structures that drive the evolution of networks may benefit the identification of other potential host determinants governing its biological phenomenon. An immediate direction and a long-term outlook for network biology should focus on algorithmic and methodological improvements for network analysis to strengthen uncertainty quantification and confidence estimation in analyses, subsequently gaining better insights into interpretability in network biology. For example, network embedding—be it via large language models or deep learning methods (see “Future perspectives”)—has garnered increasing attention in recent years.

## Concluding remarks

Current ART suppresses HIV replication. The obstacle to a functional cure for HIV eradication is due to a lack of appropriate biomarkers that allow for the detection of the precise location of reservoir cells with latent HIV infections. Given that HIV/AIDS disease progress associated with ART is dynamic, available approaches are used for the discovery of potential biomarkers; therefore, enabling the precise targeting of the microenvironment of latent HIV reservoirs is imperative.

Network-based analysis offers a window of opportunity to access such complex omics datasets and better understand the complexity of biological phenomena. The question of whether a relationship exists between structure and functionality has attracted attention. In recent years, remarkable advances in both theoretical principles and analytical tools have been made, bringing the architectural features of networks closer to the associated functional annotations. Given that the architectural features of topology, robustness, and assigned function are profoundly interlinked and can change over time, it is crucial to additionally comprehend biological networks from a temporal aspect in future research. Furthermore, the application of network-based analyses on multi-omics datasets to identify the core–peripheral structures of a network and potential personalized hub clusters across individuals may contribute to precision diagnosis and disease prognosis, allowing more flexible matching of an individual’s case with a specific treatment and dosing regimen.

This review highlighted recent HIV studies that have integrated graph theory and network-based analyses. In fact, despite intensive studies on latent HIV reservoirs over the past 40 years, numerous critical issues regarding latent or deeply latent reservoirs persist. It is possible that multidisciplinary investigations coupled with mathematical approaches can serve as potential solutions for a better understanding of the pathogenesis of this virus, as well as for other emerging infectious diseases. Of note, although immunity, cellular molecules and soluble factors, and viral DNA/RNA are the candidates of longitudinal biomarkers frequently discussed, one cannot exclude the presence of other factors that may be more adequate to serve as potential longitudinal biomarkers for HIV reservoirs.

### Future perspectives

HIV evolves at the epidemiological level and within hosts, augmenting a broader range of variability across HIV-infected individuals beyond our expectations. The clinical observation that HLA-B*57 cannot be identified in every elite controller (see Box 2) has already offered us a good example. Hence, perhaps more innovative therapeutic strategies, enabling the precise diagnosis of individuals and providing personalized treatment regimens, will be optimal for HIV eradication in the future. This review leverages the potential of the joint method, consisting of network-based analyses and mathematical-based modeling for advancements in precision medicine for the longitudinal tracking of the configuration of HIV reservoirs and capturing the distinctions in potential biomarkers targeting HIV reservoirs alongside HIV infections. In the near future, the emphasis should be placed on how to effectively collect pertinent data from individuals, followed by the establishment of cohort databases of personalized information that require the creation of “molecule profiles” of individuals, advancing not only antiretroviral therapy regimens but also predictive health.

Lastly, the modern concept of network biology has been further implemented by mingling deep learning-based approaches with network science. Such a hybrid setting of technologies is known to be increasingly feasible and particularly crucial for forthcoming biomedical applications, including drug discovery, precision medicine, disease modeling, and predictive health. For example, digital twins, which are defined as ‘‘a set of virtual information constructs that fully describes a potential or actual physical manufactured product from the microatomic level to the macro geometrical level” [108] have been applied to precision medicine [109]. Different from conventional methods in network biology that have awareness of a bias, exclusively focusing on the vertices and edges, advanced network biology should pay more attention to substantial exchanges between outputs from deep learning-driven biological networks and real-world concepts, perhaps achieving more reliable interpretability.

Box 1 Structuring a simple graph and the components involved *Vertices* are concatenated by *edges*, allowing for movement from one *vertex* to another. Such a trajectory between two *vertices* $i$ and $j$ is called *a walk,* and the *vertices* $i$ and $j$ are the endpoints of this walk (Fig. 1g). In the circumstance that every *vertex* connected by a sequence of *edges* appears only one time, this trajectory is called *a path*. No matter whether it is *a walk* or *a path*, it describes a directional movement from one *vertex* $i$ to another $j$, meaning that *edges* can be directional. A graph in which the *edges* possess directions is called a *digraph* (Fig. 1b). A *digraph* is “connected” if it cannot be separated into the union of two *digraphs* and is “strongly connected” only if a *path* always exists for any two *vertices* in the *digraph*. Given that *edges* are directional in *digraphs*, enabling one to mark a specified starting point (its “source”) and a specified ending point (its “target”), important derivatives of *digraphs* have been put forward and applied in a variety of practical situations. In this Review, I briefly describe a simple application to the study of *Markovian stochastic processes* (see the main text and *Glossary*).

A *disconnected graph* refers to a graph consisting of two or more pieces (Fig. 1c). A *planar graph* is a graph that can be drawn in a plane, in which no two *edges* geometrically intersect (Fig. 1d). Although only graphs in a two-dimensional projection are discussed here, it is worth noting that planar networks can sufficiently describe most spatial networks. A *forest* is a graph that contains no cycles, and a connected *forest* is a *tree* (Fig. 1f). In many applications, *trees* can have a hierarchical level in the setting where *vertices* are arranged to be represented as being above, below, or at the same level as other *vertices*, and it becomes essential to search the *tree* in a systematic way in case a particular piece of information is required. An analog of a *tree* has been implemented in a machine learning approach—namely, the “decision tree” [110]—which can be utilized for data mining, classification, and regression applications.

Box 2 The potential mechanism of elite control revolves around the host factors and the host genome The discovery of “elite controllers”, a subset of HIV-infected individuals who are distinguished by their ability to maintain a state of apparently durable control of HIV replication without the need for antiviral therapy [111, 112], evidences the profound involvement of host-associated factors in governing HIV pathogenesis. Remarkably, clinical studies have reported that as both patients are HLA-B*57-positive [72, 74, 113, 114], the transmitter progressed to AIDS, whereas the recipient, who is also HLA-B*57-positive, is an elite controller [115], suggesting that most likely viral determinants play only a minor role in this specific phenotype of elite control. However, it is important to note that despite the over-representation of the HLA-B*57 allele in cohorts of elite controllers, not every elite controller is HLA-B*57-positive [115–117]. Indeed, many HLA-B*57-positive patients can still be viremic and develop progressive disease [113], implying that other host-related mechanisms are responsible for silencing HIV gene expression as well. A study by Jiang et al. [101] has underscored the impact of heterochromatin associated with epigenetic features on the HIV deep latency in elite controllers. Overall, a better grasp of the functionality of the host genome may aid in comprehending the fundamental mechanism of elite control. Other clinical characteristics of elite controllers include the strong HIV-specific CD8 T cell response, the relatively small size of the reservoir, and the increased abundance of clonally expanded latent cells [118].

## Data Availability

Not applicable.

## Glossary

Markovian stochastic processes: A system where transitions between discrete states occur with probabilities, and the transition probabilities depend only on the previous state, making it memoryless [107].
Simple graph: A graph without loops and multiple *edges* (see Fig. 1a).
Directed graph/digraph: A graph in which the *edges* have a direction—for every pair of two *vertices*, one is called the “source” and another is called the “target” (see Fig. 1b).
Disconnected graph: A graph, in which at least one pair of *vertices* that have no *path* connecting them (see Fig. 1c).
Planar graph: A graph, in which no *edges* cross each other (see Fig. 1d).
Forest: A graph that contains no cycles (see Fig. 1e).
Tree: A graph that contains connected *forests* (see Fig. 1f).
Vertex (node): A *vertex* (or *node*) in a graph is one of the objects that are connected together (see Fig. 1a, the definition of *V*).
Edge: An *edge* in a graph is one of the connections between the v*ertices* (see Fig. 1a, the definition of *E*).
A walk: A sequence of *vertices* and *edges* of a graph (see Fig. 1g).
A path: A *walk*, in which no identical *vertex* appears more than once (see Fig. 1g).
Small-world property: A network system can be highly clustered, like a regular network, yet have small characteristic *path* lengths, like a random graph [47].
Scale-free property: A network system, in which degree distribution approximates a power law, *P*(*k*) ~ *k* –γ, where γ is the degree exponent and ~ indicates “proportional to” [48].
Self-similarity property: A scale-invariant renormalization procedure for dissimilar complex networks [49].
Ultra-small-world properties: The property of *scale-free* networks with degree exponents 2 < γ < 3 (γ denotes the degree exponent), a range that is observed in most biological and non-biological networks [119, 120], with the average *path* length following $l$ ~ log log N, which is significantly shorter than log N that characterizes random *small-world* networks.
Assortativity: The *assortativity* coefficient is positive and the tendency for *vertices* to connect to others with similar properties within a network [52].
Disassortativity: The *assortativity* coefficient is negative and the tendency for *vertices* to connect to others with distinct properties within a network [52].
Graphlet: A set of *vertices* defined as small induced subgraphs [56].
Bipartite graph: A graph, which can be split into two disjoint sets of *vertices* (see Fig. 1h).
Preferential attachment: A process in which *vertices* prefer to connect to others that already have many links [48].
Modularity: It is a measure representing the strength of the division of a network into modules.
